# Enhancer variants associated with Alzheimer’s disease affect gene expression via chromatin looping

**DOI:** 10.1186/s12920-019-0574-8

**Published:** 2019-09-09

**Authors:** Masataka Kikuchi, Norikazu Hara, Mai Hasegawa, Akinori Miyashita, Ryozo Kuwano, Takeshi Ikeuchi, Akihiro Nakaya

**Affiliations:** 1 Department of Genome Informatics, Graduate School of Medicine, Osaka University, 2-2 Yamadaoka, Suita, Osaka, 565-0871 Japan; 2 Department of Molecular Genetics, Brain Research Institute, Niigata University, Niigata, Japan; 3 Asahigawaso Medical-Welfare Center, Asahigawaso Research Institute, Okayama, Japan

**Keywords:** Alzheimer’s disease, Genome-wide association study, Non-coding variants, Chromatin higher-order structure

## Abstract

**Background:**

Genome-wide association studies (GWASs) have identified single-nucleotide polymorphisms (SNPs) that may be genetic factors underlying Alzheimer’s disease (AD). However, how these AD-associated SNPs (AD SNPs) contribute to the pathogenesis of this disease is poorly understood because most of them are located in non-coding regions, such as introns and intergenic regions. Previous studies reported that some disease-associated SNPs affect regulatory elements including enhancers. We hypothesized that non-coding AD SNPs are located in enhancers and affect gene expression levels via chromatin loops.

**Methods:**

To characterize AD SNPs within non-coding regions, we extracted 406 AD SNPs with GWAS *p*-values of less than 1.00 × 10^− 6^ from the GWAS catalog database. Of these, we selected 392 SNPs within non-coding regions. Next, we checked whether those non-coding AD SNPs were located in enhancers that typically regulate gene expression levels using publicly available data for enhancers that were predicted in 127 human tissues or cell types. We sought expression quantitative trait locus (eQTL) genes affected by non-coding AD SNPs within enhancers because enhancers are regulatory elements that influence the gene expression levels. To elucidate how the non-coding AD SNPs within enhancers affect the gene expression levels, we identified chromatin-chromatin interactions by Hi-C experiments.

**Results:**

We report the following findings: (1) nearly 30% of non-coding AD SNPs are located in enhancers; (2) eQTL genes affected by non-coding AD SNPs within enhancers are associated with amyloid beta clearance, synaptic transmission, and immune responses; (3) 95% of the AD SNPs located in enhancers co-localize with their eQTL genes in topologically associating domains suggesting that regulation may occur through chromatin higher-order structures; (4) rs1476679 spatially contacts the promoters of eQTL genes via CTCF-CTCF interactions; (5) the effect of other AD SNPs such as rs7364180 is likely to be, at least in part, indirect through regulation of transcription factors that in turn regulate AD associated genes.

**Conclusion:**

Our results suggest that non-coding AD SNPs may affect the function of enhancers thereby influencing the expression levels of surrounding or distant genes via chromatin loops. This result may explain how some non-coding AD SNPs contribute to AD pathogenesis.

**Electronic supplementary material:**

The online version of this article (10.1186/s12920-019-0574-8) contains supplementary material, which is available to authorized users.

## Background

Alzheimer’s disease (AD) is a neurodegenerative disease characterized by cognitive impairment. In postmortem brains from AD patients, amyloid beta (Aβ) deposits on the surface of neurons and intracellular aggregations of hyperphosphorylated tau protein are observed. The heritability of AD is estimated to be between 58 and 79% [1]. The *APOE* ε4 allele is the genetic factor with the strongest influence identified to date on the risk of late-onset AD (LOAD). Genome-wide association studies (GWASs) have found 20 genetic loci associated with AD [2–8]. Furthermore, genome-wide linkage studies have reported the associations with 14 genomic regions, including nearly 150 genes [9]. Of them, *APOE*, *CLU*, *CR1*, and *PICALM* were found in both of the GWASs and the linkage studies. *CLU* is located in a chromosome 8p21.1 and encodes clusterin or apolipoprotein J. *CR1* is located in a chromosome 1q32.2 gene and encodes the complement component (3b/4b) receptor 1. *CLU* and *CR1* are associated with immune and inflammatory responses. *PICALM* is located in a chromosome q14.2 and encodes phosphatidylinositol binding clathrin assembly protein. *PICALM* is involved in clathrin-mediated endocytosis. Most of the genes that were reported in the GWASs including above genes are the closest genes of the single-nucleotide polymorphisms (SNPs) identified in these GWASs, however, it is unclear whether the GWAS SNPs really affect the closest genes because most of the GWAS SNPs are located in non-coding regions, such as introns and intergenic regions. In fact, 98% of the SNPs that were found in a recent GWAS meta-analysis were located in non-coding regions [10]. These AD-associated SNPs (AD SNPs) could be tag SNPs of surrounding functional exonic variants [11]; however, a fine-mapping study of *BIN1*, *CLU*, *CR1*, and *PICALM*, which are the closest genes to several AD SNPs, showed no direct association with AD pathogenesis [12].

Recent studies have reported that disease-associated non-coding SNPs alter the functions of regulatory sequences, such as enhancers that typically regulate gene expression levels. For instance, Soldner et al. showed that a non-coding risk variant rs356168, which is associated with Parkinson’s disease (PD), is located in an enhancer region and upregulates the expression level of a PD-susceptibility gene *SNCA* [13]. It is reported that some SNPs in AD influenced gene expression levels as in AD [14, 15]. In particular, Karch et al. searched functional AD SNPs from 21 loci that were found in the IGAP GWAS and revealed that the *ZCWPW1* and the *CELF1* loci were associated with some gene expressions [15].

The SNPs that influence gene expression levels as mentioned above are called expression quantitative trait loci (eQTLs). eQTLs are useful for considering function of non-coding SNPs, however this approach only achieves indirect evidence because eQTL effects are usually determined by correlations between genotypes and expression levels of target genes [16]. One of the molecular mechanisms to explain eQTL effects is contact between eQTLs and target genes by the formation of chromatin loops. Chromatin regions including eQTLs fold in order to bring in proximity to the genes they regulate. A growing body of evidence indicates that disease-associated variants in enhancers affect the expression levels of distal genes via chromatin loops in several diseases such as frontotemporal lobar degeneration, which belongs to the group of neurodegenerative diseases that includes AD [17–20]. These findings suggest that non-coding AD SNPs may alter the functions of regulatory sequences, such as enhancers that typically regulate gene expression levels via chromatin loops. Thus, we hypothesized that non-coding AD SNPs are located in enhancers and affect gene expression levels.

To test this hypothesis, we analyzed 392 AD SNPs located in non-coding regions by integrating enhancer activity data and chromatin interaction data. In particular, we used data from the Encyclopedia of DNA Elements (ENCODE) project [21] and the Roadmap Epigenomics project [22]. These projects measured epigenomic markers, including histone modifications and DNase I-hypersensitive sites, across every human tissue or cell type, and used these data to estimate genome-wide chromatin states (e.g., whether an enhancer is activated or not) [23, 24]. To identify chromatin–chromatin interactions such as chromatin loops, we used data from the chromosome conformation capture (3C) variant Hi-C, which can capture genome-wide chromatin interactions via high-throughput sequencing. We found that nearly 30% of the non-coding AD SNPs were located in enhancers and that they affected the expression of genes associated with Aβ clearance, synaptic transmission, and immune responses. Among the non-coding AD SNPs, rs1476679 at the *ZCWPW1* gene locus and rs7364180 at the *CCDC134* gene locus were associated with several eQTL genes, which are the genes influenced by the eQTLs. Finally, analysis of chromatin higher-order structure revealed direct associations between rs1476679 and eQTL genes. Our findings would explain the regulatory mechanism of this AD SNP.

## Methods

### AD-associated SNPs (AD SNPs)

AD SNPs were obtained from the GWAS catalog database (Release 20,170,627, ftp://ftp.ebi.ac.uk/pub/databases/gwas/releases/2017/06/27/gwas-catalog-associations.tsv) [25]. These SNPs included “Alzheimer” in the “DISEASE/TRAIT” column of the GWAS catalog data. We investigated 406 of these SNPs, including 19 confirmed SNPs identified in the IGAP study [10] and AD SNPs with GWAS *p*-values of less than 1.00 × 10^− 6^, which is used as a suggested threshold in GWAS. The suggested threshold is generally used as a common threshold for initial SNP selection in many studies. The SNPs that satisfied a GWAS suggestive threshold are not fully denied the association with diseases. The statistical significance of those SNPs may increase with an increase in sample size or meta-analysis. In this study, we analyzed the SNPs that are likely to relate to AD, not just the SNPs that were strongly associated with AD. The non-coding AD SNPs and the methods for association test are described in Additional file 1: Table S1. The genomic positions of all SNPs were standardized to the human reference genome (hg19) based on their reference SNP ID (rsID). SNPs without a rsID were manually curated.

### Enhancer data from 127 tissues or cell types

A chromatin state model for 127 tissues or cell types was obtained from the Roadmap Epigenomics website (http://egg2.wustl.edu/roadmap/web_portal/). These 127 tissues or cell types are described in Additional file 1: Table S2. The chromatin state model segments the human genome into 25 states based on 12 chromatin marks (H3K4me1, H3K4me2, H3K4me3, H3K9ac, H3K27ac, H4K20me1, H3K79me2, H3K36me3, H3K9me3, H3K27me3, H2A.Z, and DNase I-hypersensitive sites) using ChromHMM and ChromImpute [23, 24]. We extracted six enhancer states (Active Enhancer 1 (EnhA1), Active Enhancer 2 (EnhA2), Active Enhancer Flank (EnhAF), Weak Enhancer 1 (EnhW1), Weak Enhancer 2 (EnhW2), and Primary H3K27ac possible Enhancer (EnhAc)) from the 25 states and treated them as enhancer data (Additional file 1: Table S3). EnhA1, EnhA2, and EnhAF show high levels of H3K4me1 and H3K27ac, which are enhancer-associated histone modifications. EnhW1 and EnhW2 show high H3K4me1 and low H3K27ac levels. EnhAc shows low H3K4me1 and high H3K27ac levels.

### Expression quantitative trait loci (eQTLs)

The eQTL genes of each AD SNP were searched in the GTEx Portal database (https://www.gtexportal.org/) [26, 27] and the BRAINEAC database (http://www.braineac.org/) [28]. For further details, see Additional file 2: Supplementary Information. The eQTL genes in the above databases are located on the same chromosome as the associated SNPs. Pseudogenes were removed based on the GENCODE pseudogene resource from the eQTL analysis [29]. The AD SNPs were considered to associate with the eQTL genes if the corrected *p*-value was less than 0.05. Each p-value was corrected for multiple testing across genes on the same chromosome using Storey’s q-value [30]. Gene functional enrichment analysis of the eQTL genes was performed using the Metascape database (http://metascape.org/) [31].

### Differentially expressed genes (DEGs) from publicly available datasets

DEGs between AD and non-demented brains were identified using three publicly available gene expression datasets (syn5550404 [32], GSE5281 [33], and GSE44770 [34]). For further details, see Additional file 2: Supplementary Information. The syn5550404 dataset contains RNA-seq data for cerebellum and temporal cortex samples from 312 Caucasian subjects with neuropathological diagnosis of AD, progressive supranuclear palsy, pathologic aging or elderly controls without neurodegenerative diseases. The DEGs were identified using multivariate linear regression after adjusting for covariates (age at death, gender, RNA integrity number (RIN), source, and flow cell). These statistics were provided by the AMP-AD Knowledge Portal (https://www.synapse.org/#!Synapse:syn2580853/wiki/409840). The GSE5281 dataset contains microarray data for six brain regions that are either histopathologically or metabolically relevant to 33 AD and 14 aging; these include the entorhinal cortex (BA 28 and 34), superior frontal gyrus (BA 10 and 11 and approximate BA 8), hippocampus, primary visual cortex (BA 17), middle temporal gyrus (BA 21 and 37 and approximate BA 22), and the posterior cingulate cortex (BA 23 and 31). The GSE44770 dataset contains microarray data for 230 autopsied tissues from dorsolateral prefrontal cortex (PFC), visual cortex (VC) and cerebellum (CR) in brains of LOAD patients, and non-demented healthy controls. These two datasets were reanalyzed, because statistics for the comparisons were not provided. The reanalyses of GSE5281 and GSE44770 was performed using t-tests and logistic regression analyses with covariates (age, gender, postmortem interval in hours, sample pH, RIN, sample processing, and batch), respectively, as described in the original analyses. DEGs were defined based on an FDR-adjusted *p*-value < 0.05.

### Overlap between the eQTL genes and the DEGs

To test whether the DEGs significantly include the eQTL genes, we calculated the p-value by hypergeometric distribution test and the fold enrichment ratio (FER) as follows:

$$ P\left(X=x\right)=\frac{(mx)\left(N- mn-x\right)}{(Nn)}, $$

$$ FER=\frac{x}{E}, $$

$$ E=\frac{mn}{N}, $$

where *x* is the number of genes which are the DEGs and the eQTL genes, and *m* and *n* are the numbers of the DEGs and the eQTL genes, respectively. *P(x)* is a probability when the number of genes which are the DEGs and the eQTL genes is *x*. *N* is the total number of genes that were analyzed in the dataset. *E* is the expected value. When the FER was greater than 1, the overlap between the DEGs and the eQTL genes was higher than an expected value.

### Cell culture

We employed two cell lines for this study: the neuroblastoma cell line SK-N-SH (American Type Culture Collection, Manassas, VA, USA) (HTB-11) and the astrocytoma cell line U-251 MG (Japan Collection of Research Bioresources Cell Bank, Ibaraki, Osaka, Japan) (IFO50288). Both cell lines were grown in Eagle’s MEM and cultured at 37 °C with 5% CO_2_. For further details, see Additional file 2: Supplementary Information.

### TCC library preparation and deep sequencing for target regions

Tethered conformation capture (TCC), which is a variation of Hi-C, was performed to detect chromatin interactions. A TCC library was prepared in accordance with the method reported by Kalhor et al. with minor modifications [35]. The captured DNA fragments corresponding to the target regions were obtained from the TCC library using the SureSelect Target Enrichment System (Agilent Technologies). The library was subjected to paired-end sequencing on the Genome Analyzer IIx or MiSeq (Illumina) platform. For further details, see Additional file 2: Supplementary Information.

### Processing the sequencing output

In accordance with the procedure established by Imakaev et al. [36], we mapped the sequenced reads to the human reference genome (hg19) using Bowtie2 and used the “hiclib” library (provided by the Leonid Mirny Laboratory (https://bitbucket.org/mirnylab/)) to filter out non-informative reads. For further details, see Additional file 2: Supplementary Information.

### Chromatin interaction analysis

To identify significant chromatin interactions, we applied the R software package fourSig [37]. We counted mapped reads from TCC to the nearest restriction sites (HindIII sites) because TCC assumes that chromatin interactions occur around restriction sites. A viewpoint nearest to the HindIII sites on both sides of the SNP was selected when we detected chromatin interactions for an SNP. A window size of one fragment was set. The significance level employed was an FDR-adjusted *p*-value of 0.05.

### Identification of topologically associating domains (TADs)

Identification of TADs in the SK-N-SH and U-251MG cell lines was performed using the R software packages HiCdat and HiCseg [38, 39]. The sequenced reads mapped to the human reference genome (hg19) were normalized using HiCdat and compiled using a genomic bin size of 100 kb. The default values for the HiCseg parameters were employed to detect TADs. HiCseg estimated the TAD block boundaries based on a maximum likelihood approach.

## Results

### Nearly 30% of non-coding AD SNPs are located in enhancers

**Fig. 1:**
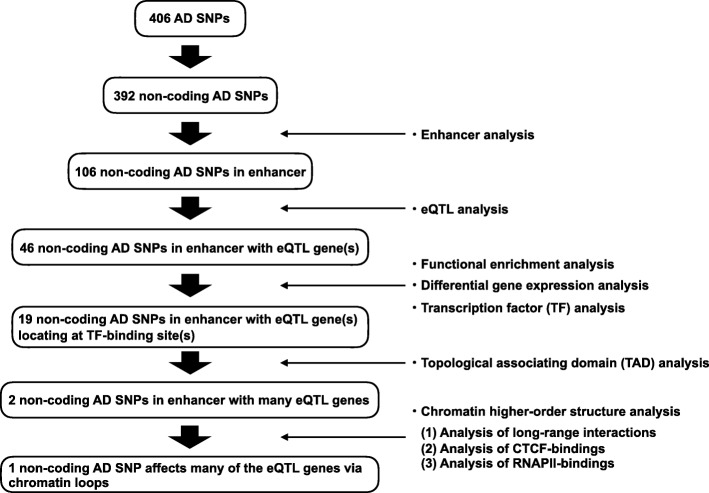
Flowchart of the present study

**Fig. 2:**
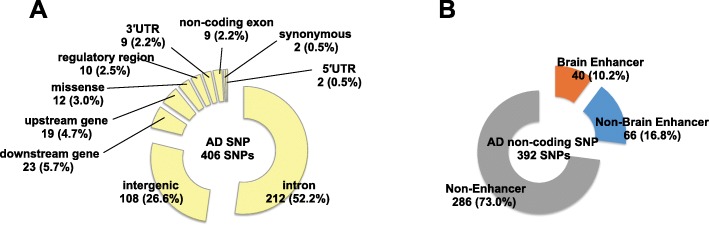
Nearly 30% of non-coding AD SNPs are located in enhancers. **a** Circle chart showing the genomic region location of AD SNPs from the GWAS catalog database (*p*-value < 1.00 × 10^− 6^). **b** Circle chart showing the proportions of non-coding AD SNPs located in non-enhancer regions and in enhancers identified in one or more tissues or cell types. “Brain Enhancer” indicates non-coding AD SNPs located in enhancers identified in one or more brain tissues. “Non-Brain Enhancer” indicates non-coding AD SNPs located in enhancers that were not identified in brain tissues but were identified in the other tissues or cell types. All tissue and cell type names are described in Additional file 1: Table S2

Figure 1 provides an overview of our study. First, we collected AD SNPs from the GWAS catalog database [25]. These AD SNPs have GWAS *p*-values of less than 1.00 × 10^− 6^, which is used as a suggested threshold in GWAS. Among the 406 AD SNPs, 392 SNPs (96.6%) were in non-coding regions, whereas the rest were missense and synonymous mutations (Fig. 2a). Next, we checked whether these non-coding AD SNPs were located in enhancers, using publicly available enhancer data. Enhancer locations were predicted based on 11 histone modifications and DNase I-hypersensitive sites quantified in 127 human tissues or cell types, including 10 brain tissues (see Methods). We counted non-coding AD SNPs located in the enhancers that were predicted in one or more tissues or cell types. Among the 392 non-coding AD SNPs, 106 (27.0%) were in enhancers (Fig. 2b). Of these 106 SNPs, 40 (10.2% of the 392 non-coding AD SNPs) were in enhancers identified in one or more brain tissues.

### Genes affected by non-coding AD SNPs are related to AD-relevant processes and are often differentially expressed in AD patients

The 106 non-coding AD SNPs may affect the expression levels of genes in any tissue or cell type because enhancers are regulatory elements that influence the expression levels of genes. Next, we investigated whether the non-coding AD SNPs functioned as eQTLs, which affect gene expression levels. To this end, the genes influenced by the non-coding AD SNPs (hereafter referred to as eQTL genes) were collected from the GTEx Portal [26, 27] and BRAINEAC databases [28]. We used the eQTL genes that are located on the same chromosome as the associated AD SNPs. Among the 106 non-coding AD SNPs located in enhancers, 46 SNPs were associated with at least one eQTL gene and, overall, 130 eQTL genes were identified. These eQTL genes were related to Aβ formation, synaptic transmission, and immune responses (Table 1). These results were replicated even when different databases were used (Additional file 1: Tables S4 and S5). Interestingly, AD GWAS SNPs from a previous GWAS meta-analysis study were also associated with immune responses [40].

**Table 1:** Gene functional enrichment analysis

GO	Category	Description	Count	%	Log10(P)	Log10(q)
GO:1902430	GO Biological Processes	Negative regulation of amyloid-beta formation	3	2.38	-5.03	-0.94
GO:0007271	GO Biological Processes	Synaptic transmission, cholinergic	4	3.17	-4.47	-0.91
R-HSA-1834949	Reactome Gene Sets	Cytosolic sensors of pathogen-associated DNA	5	3.97	-4.34	-0.89
GO:0002768	GO Biological Processes	Immune response-regulating cell surface receptor signaling pathway	11	8.73	-4.29	-0.89
GO:0072665	GO Biological Processes	Protein localization to vacuole	4	3.17	-3.86	-0.8
GO:0006353	GO Biological Processes	DNA-templated transcription, termination	5	3.97	-3.45	-0.59
GO:0016032	GO Biological Processes	Viral process	13	10.32	-3.35	-0.53
GO:0007169	GO Biological Processes	Transmembrane receptor protein tyrosine kinase signaling pathway	11	8.73	-2.74	-0.26
GO:0071466	GO Biological Processes	Cellular response to xenobiotic stimulus	5	3.97	-2.62	-0.2
GO:0007127	GO Biological Processes	Meiosis I	4	3.17	-2.36	-0.02
GO:0031329	GO Biological Processes	Regulation of cellular catabolic process	11	8.73	-2.33	-0.02
R-HSA-5653656	Reactome Gene Sets	Vesicle-mediated transport	10	7.94	-2.31	-0.01
M254	Canonical Pathways	PID MYC REPRESS PATHWAY	3	2.38	-2.27	0
GO:0043547	GO Biological Processes	Positive regulation of GTPase activity	7	5.56	-2.09	0
GO:0002274	GO Biological Processes	Myeloid leukocyte activation	9	7.14	-2.06	0

Terms with *p*-value < 0.01, minimum count 3, and enrichment factor > 1.5 (enrichment factor is the ratio between observed count and the count expected by chance) are collected and grouped into clusters based on their membership similarities

We tested whether the eQTL genes were differentially expressed between AD and non-AD brain tissues. For this analysis, we used three publicly available datasets (syn5550404 [32], GSE5281 [33], and GSE44770 [34]) that include gene expression data analyzed in nine human brain tissues (prefrontal cortex, temporal cortex, visual cortex, entorhinal cortex, hippocampus, medial temporal gyrus, posterior cingulate, superior frontal gyrus, and cerebellum). Differentially expressed genes (DEGs) between AD and non-AD were identified in each brain tissue in each dataset (FDR < 0.05) (Additional file 1: Table S6)). We counted the number of the eQTL genes that were identified as the DEGs and test whether the DEGs significantly include the eQTL genes using a hypergeometric distribution test (see Methods). Our results showed that the eQTL genes were significantly included among the DEGs in some tissues including the entorhinal cortex and hippocampus that are closely related to AD pathologies (Table 2). Among the 126 eQTL genes analyzed in these datasets (4 of the 130 eQTL genes were not analyzed in the datasets because those corresponding probe sets were not constructed in the microarray or those transcripts did not satisfy criteria in RNA-seq), 110 genes (87.3%) were differentially expressed in one or more brain tissues or datasets. Additionally, 35 of 46 SNPs (76.1%) had one or more these differentially expressed eQTL genes. These results suggested that the non-coding AD SNPs affected genes whose expression levels were altered in the AD brain.

**Table 2:**
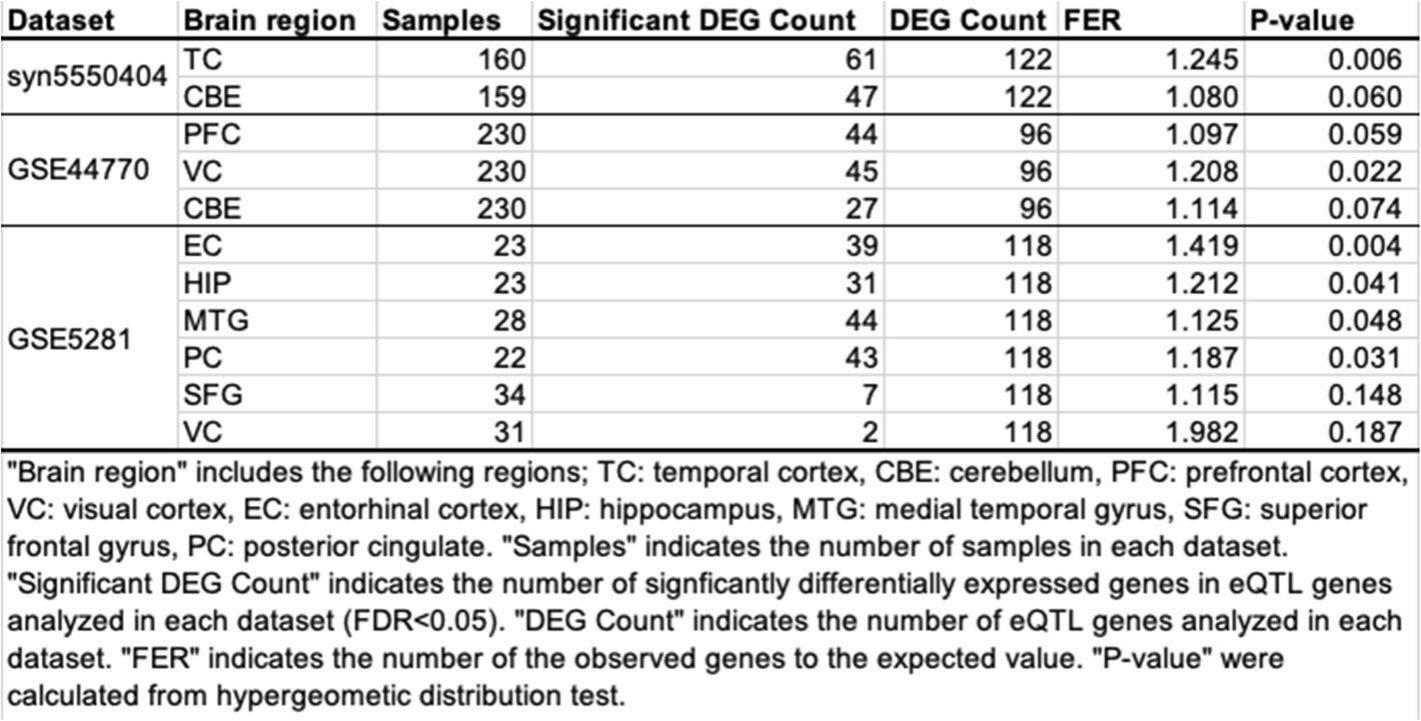
Statistical test for DEG enrichment in eQTL genes

“Brain region” includes the following regions; *TC* temporal cortex, *CBE* cerebellum, *PFC* prefrontal cortex, *VC* visual cortex, *EC* entorhinal cortex, *HIP* hippocampus, *MTG* medial temporal gyrus, *SFG* superior frontal gyrus, *PC* posterior cingulate. “Samples” indicates the number of samples in each dataset. “Significant DEG Count” indicates the number of significantly differentially expressed genes in eQTL genes dataset. “FER” indicates the number of the observed genes to the expected value. “*P*-value” were calculated from hypergeometic distribution test

### AD SNPs with eQTL effects are often located at protein-binding sites

Enhancers are regulatory regions that control the expression levels of surrounding genes when bound by specific proteins, such as transcription factors (TFs). To emphasize that the non-coding AD SNPs are located in the enhancers, we looked for TF-binding sites in these enhancers using the ENCODE ChIP-seq data for 161 TFs from 91 human cell types, which included 17 brain tissues or cell types (Additional file 1: Table S7). Among the 46 SNPs with eQTL effects discussed above, 19 were located at a protein-binding site in at least one cell types (Table 3). The closest genes were the corresponding eQTL genes for only eight of these SNPs, indicating that GWAS SNPs do not always affect the closest genes (Table 3). Four SNPs of the SNPs (rs4663105, rs1532278, rs4147929, and rs439401) were located around well-known AD candidate genes (*BIN1*, *CLU*, *ABCA7*, and *APOE*) and were eQTLs of those genes. The *BIN1* locus rs4663105 was located in enhancer that was activated in five tissues or cell types. Interestingly, all of these tissues or cell types were from brain regions including the hippocampus, suggesting that rs4663105 has the brain-specific eQTL effects (Additional file 1: Table S8). An enhancer near *CLU* locus was activated in 63 tissues or cell types including 4 brain tissues. The *APOE* locus rs439401 is located in the *APOE*-*APOC1* intergenic region. Enhancers near rs439401 were activated in 102 tissues or cell types including 7 brain tissues (Additional file 1: Table S8). This region is known as multienhancer 1 and affects *APOE* expression in various tissues or cell types, including macrophages, adipose tissue, and a neuronal cell line [41, 42]. Indeed, *APOE* was identified as one eQTL gene of rs439401 in our study. On the other hand, 28 tissues or cell types where the enhancer involving the *ABCA7* locus was activated did not include brain tissues and were mainly from immune cells, such as monocytes, B cells, and T cells.

**Table 3:**
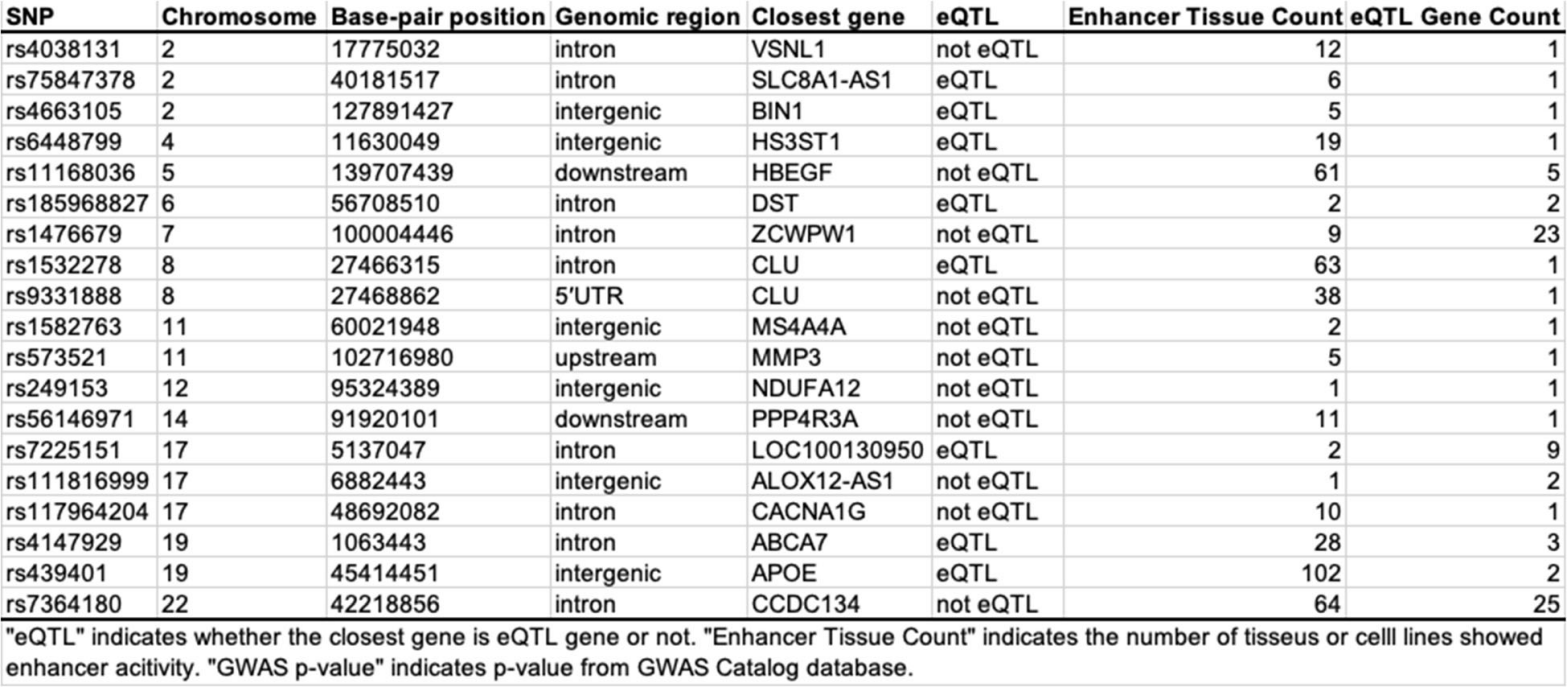
List of 19 SNPs that were located at protein-binding sites and that have eQTLgene(s)

“eQTL” indicates whether the closest gene is eQTL gene or not. “Enhancer Tissue Count” indicates the number of tissues or cell lines showed enhancer activity. “GWAS *p*-value” indicates *p*-value from GWAS Catalog database

### AD SNPs and their eQTL genes co-localize in topologically associating domains

**Fig. 3:**
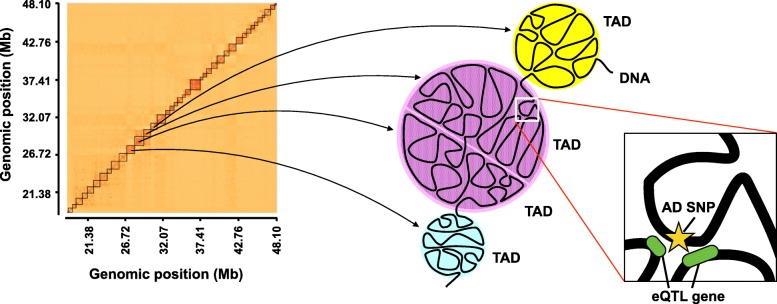
AD SNPs and their eQTL genes co-localize in topologically associating domains (TADs). Heatmap showing the frequency of chromatin interactions based on tethered conformation capture (TCC) experiments in the astrocytoma cell line U-251MG (100-kb bins). Diagonal darker blocks indicate TAD. AD SNP could contact the distal eQTL genes via chromatin interactions

A fundamental mechanism underlying the effects of eQTLs on their regulated genes is enhancer-promoter regulation via chromatin higher-order structures, such as chromatin loops. Therefore, we examined whether the non-coding AD SNPs in enhancers regulate their eQTL genes through chromatin higher-order structures. We focused on topologically associating domains (TADs), which are genomic regions that spatially interact with each other (Fig. 3) [43, 44], since enhancers and their targeted promoters aggregate in the same TAD [45, 46]. Therefore, we examined whether the 19 SNPs shown in Table 3 co-localized with the corresponding eQTL genes in the same TAD. To detect TADs, we performed tethered conformation capture (TCC), which is a variant of the Hi-C method that is used for the identification of comprehensive chromatin loops through paired-end sequencing [35]. The neuroblastoma cell line SK-N-SH and the astrocytoma cell line U-251 MG were analyzed in the TCC experiment. These cell lines were used as models of brain cells. TADs were detected in each cell line using HiCdat and HiCseg software [38, 39]. Among the 19 SNPs, 18 SNPs (94.7%) co-localized with at least one eQTL gene in the same TAD in the SK-N-SH and/or U-251 MG cell line (Table 4). Furthermore, 13 SNPs in SK-N-SH and 14 SNPs in U-251 MG co-localized in the same TAD with more than 80% of the eQTL genes associated with that particular SNP. These results suggested that the AD SNPs might regulate eQTL genes in the same TAD through chromatin higher-order structures.

**Table 4:**
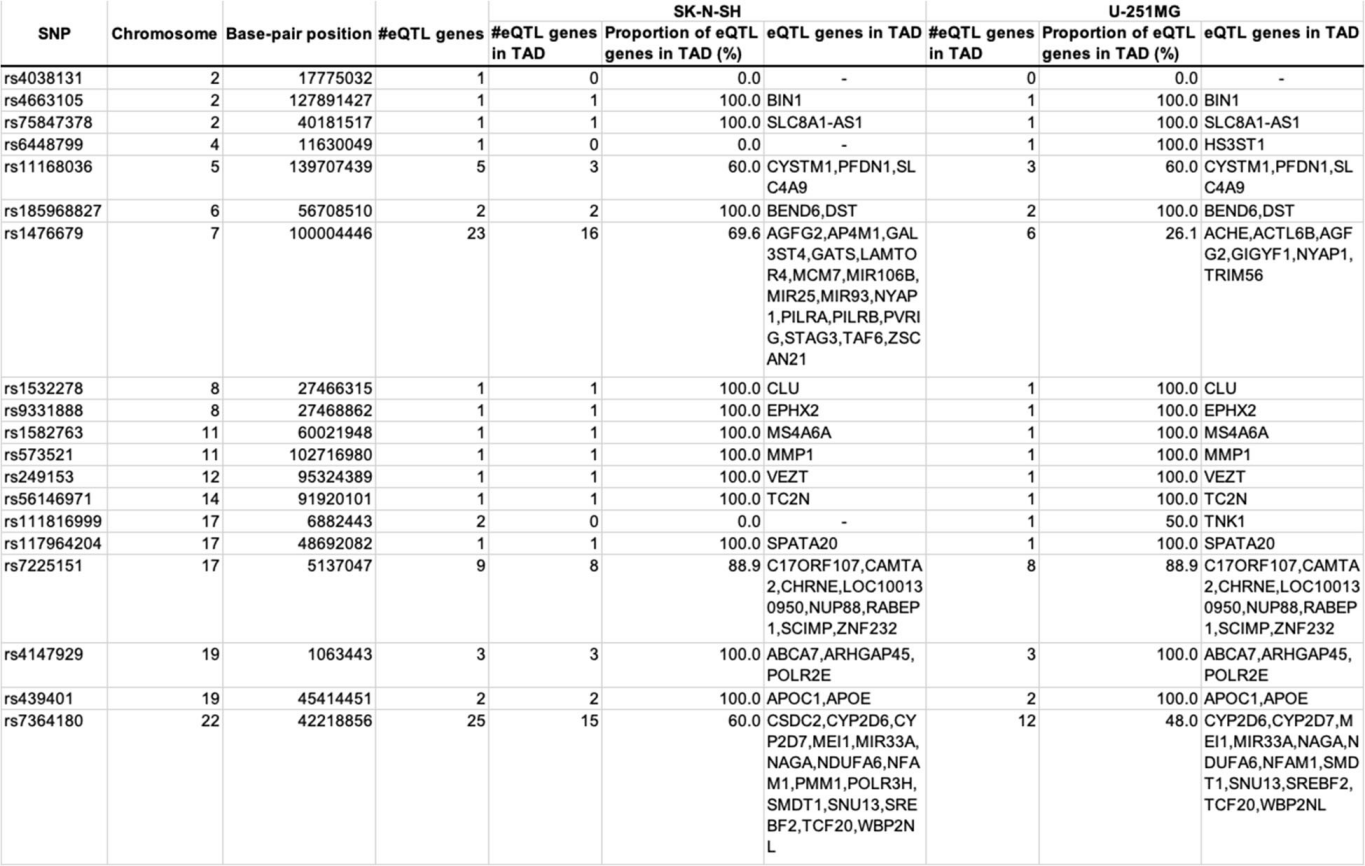
eQTL genes in TAD

### AD SNPs associating with many eQTL genes are located in CTCF-binding sites

**Fig. 4:**
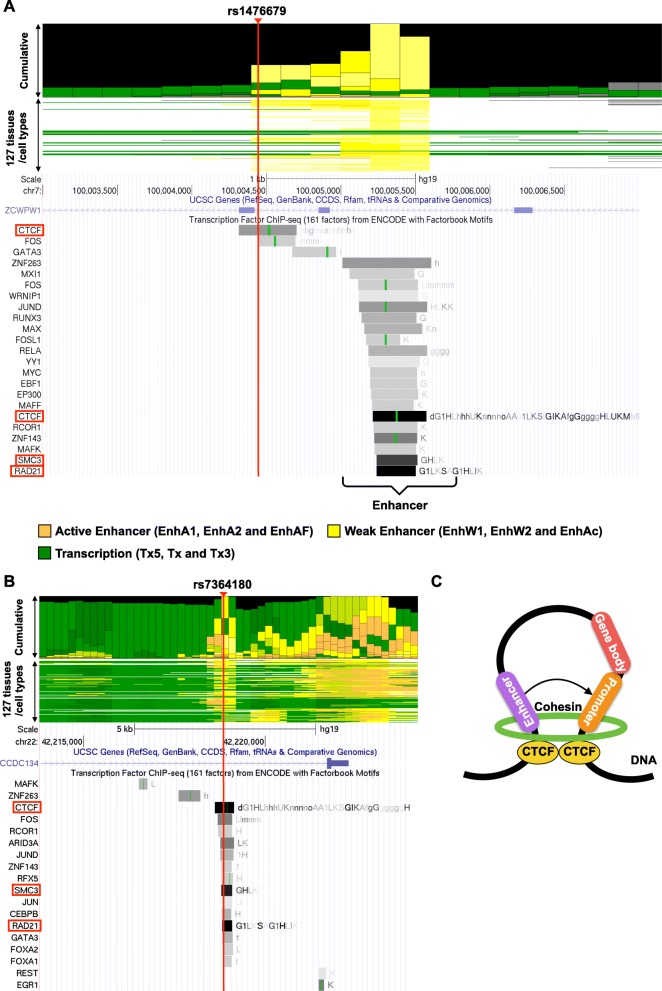
AD SNPs with eQTL effects are often located at protein-binding sites. **a**, **b** Cumulative bar graph of the chromatin state across 127 tissues or cell types (upper panel) and ChIP-seq tracks (bottom panel) around rs1476679 (**a**) and rs7364180 (**b**). Three representative chromatin state groups of the cumulative bar graph are depicted according to the color legend. The chromatin state names are shown in parentheses (see details in Methods). Details of all 25 chromatin state names are described in Additional file 1: TableS3. Grey bars in ChIP-seq tracks represent peak clusters of transcription factor (TF) occupancy. The color intensity of the bars is proportional to the level of TF occupancy. Green bars represent motif sites for the corresponding TFs. These ChIP-seq tracks were generated from the UCSC genome browser (https://genome.ucsc.edu/). **c** A schematic representation of a chromatin loop based on CTCF binding

We found that rs1476679 and rs7364180 SNPs were associated with a particularly large number of eQTL genes (23 and 25, respectively) in Table 3. rs1476679 is located in the intronic region of the *ZCWPW1* gene and in the enhancer where it is was activated in nine tissues and cell lines including adipocytes and chondrocytes, but not brain tissues (Fig. 4a and Additional file 1: Table S9). rs7364180 is located in the intronic region of the *CCDC134* gene and in the enhancer where it was activated in 64 tissues or cell types, including 5 brain tissues (Fig. 4b and Additional file 1: Table S9). These SNPs co-localized with only approximately 30–70% (depending on the cell lines) of the corresponding eQTL genes in the same TAD (Table 4), suggesting that these SNPs affected eQTL genes outside of the TADs via long-range chromatin interactions. Interestingly, rs1476679 and rs7364180 SNPs were localized at protein-binding sites of the CCCTC-binding factor (CTCF) in 12 and 66 cell lines, respectively, including neuronal cell lines (Fig. 4a, b and Additional file 1: Table S10). CTCF is a key factor to form chromatin loops and protects promoters against acting by chance from distant enhancers [47]. Chromatin loops are formed by the dimerization of two CTCF proteins binding to both regions that interact each other and the binding of a ring-shaped cohesin complex (Fig. 4c) [48–50]. The formation of chromatin loops draws enhancers closer to promoters and can influence the expression of nearby or distant genes. In addition, we found binding sites for several TFs within the 1 kb region downstream from rs1476679 (Fig. 4a) and in the region including rs7364180 (Fig. 4b). The TFs binding to these regions included SMC3 and RAD21, which are components of the cohesin complex. The enhancer region in the 1 kb region downstream from rs1476679 was activated in 63 tissues and cell lines including a brain tissue (Fig. 4a and Additional file 1: Table S9). These findings suggested that rs1476679 and rs7364180 might be involved in the formation of chromatin loops via CTCF which could regulate the expression levels of eQTL genes in cooperation with nearby enhancer regions.

### rs1476679 spatially contacts many eQTL genes via CTCF-mediated chromatin loops

To provide further insight on how rs1476679 and rs7364180 may have an effect on eQTL genes through chromatin higher-order structures, we performed the following analyses: (1) investigation of whether rs1476679 and rs7364180 displayed long-range chromatin interactions; (2) evaluation of whether the SNPs and their eQTL genes spatially contacted each other through CTCF-CTCF interactions; and (3) examination of whether RNA polymerase II (RNAPII) bound upstream of the eQTL genes interacting with the SNPs.

**Fig. 5:**
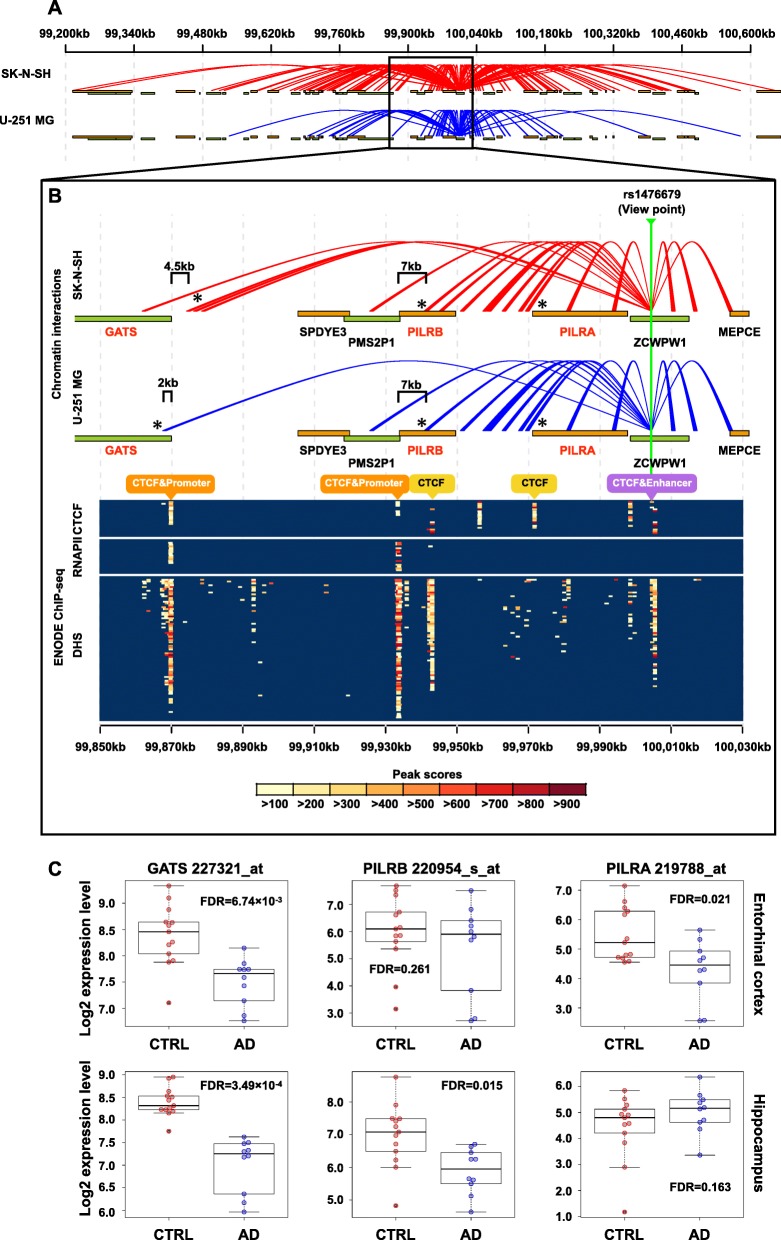
rs1476679 spatially contacts many eQTL genes via CTCF-mediated chromatin loops and affects their expression levels. **a** Chromatin interactions of the rs1476679 locus as determined by TCC experiments. Red and blue lines represent significant chromatin interactions from rs1476679 in SK-N-SH and U-251 MG cells, respectively. **b** Zoom-in region of the rs1476679 locus. The upper panel indicates chromatin interactions of the s1476679 locus. The orange and green bands indicate gene bodies on the positive and negative strands, respectively. Gene symbols in red indicate the eQTL genes of rs1476679. Asterisks indicate chromatin interactions of the rs1476679 locus with the *GATS*, *PILRB*, and *PILRA* genes. In the bottom panel, the color plot indicates the peak scores from ChIP-seq data for CTCF or RNA polymerase II (RNAPII) and DNase-seq data showing DNase I-hypersensitive sites (DHSs). Each row in the colored plot represents different brain tissues or neuronal cell lines (18 experiments (rows) including nine tissues or cell lines based on CTCF ChIP-seq, 21 experiments including 10 cell lines based on RNAPII ChIP-seq, and 82 experiments including 31 cell lines based on DNase-seq; Additional file 1: Table S11). **c** *GATS*, *PILRB*, and *PILRA* expression levels in the hippocampus and entorhinal cortex from GSE5281. Boxes represent the interquartile range between the first and third quartiles and median (internal line). Whiskers denote the lowest and highest values within 1.5 times the range of the 1 first and third quartiles, respectively; dotsrepresent *GATS*, *PILRB*, and *PILRA* expression levels in each sample

First, we investigated chromatin loops formed by rs1476679. To this end, we applied the fourSig method [37] to the TCC data from the SK-N-SH and U-251 MG cell lines. We found that the chromatin loops extended approximately ±500 kb from rs1476679 (Fig. 5a). We analyzed publicly available data and validated this extensive interacting region through chromatin interaction analysis using paired-end tag sequencing (ChIA-PET), which is experimental method used to identify chromatin loops, in the 3D Genome Browser [51] (Additional file 3: Figure S1). The identified chromatin loops were located within 5 kb of the transcription start sites (TSSs) of 15 eQTL genes associated with rs1476679 (15 out of 23 genes = 65.2%), suggesting that rs1476679 spatially contacted many of the eQTL genes through long-range chromatin interactions.

Previous eQTL studies of AD have indicated that rs1476679 was associated with *PILRB* and *GATS* gene expression levels [15, 52]; however, it is not known how rs1476679 regulates these gene expression levels. To examine whether rs1476679 contacts promoter regions of these genes via chromatin loops, we visualized significant chromatin loops with the eQTL genes *PILRB* and *GATS* genes, which were significantly downregulated in the AD hippocampus (FDR = 3.49 × 10^− 4^ for *GATS* and FDR = 0.015 for *PILRB*) or entorhinal cortex, which is affected in the early stages of AD (FDR = 6.74 × 10^− 3^ for *GATS* and FDR = 0.261 for *PILRB*) (Fig. 5b (upper panel) and Fig. 5c). In particular, the rs1476679 region significantly interacted with the gene body of *PILRB* and the upstream region of *GATS* in the two cell lines analyzed (asterisks in Fig. 5b (upper panel)). We also found chromatin loops with the eQTL gene *PILRA* gene. *PILRA* gene was significantly downregulated in the AD entorhinal cortex (FDR = 0.021) although not in hippocampus (FDR = 0.163).

Next, we investigated whether the chromatin loops between rs1476679 and *PILRA*, *PILRB* and *GATS* occurred via CTCF-CTCF interactions, which require binding of CTCF to each interacting region. To this end, we determined whether CTCF binds to the *PILRA*, *PILRB* and *GATS* loci using ChiP-seq data for CTCF in brain tissues or neuronal cell lines from the ChIP-Atlas database (nine tissues or cell lines; Additional file 1: S11). Furthermore, we used DNase-seq data to identify CTCF-binding at DNase I-hypersensitive sites (DHSs) (i.e., open-chromatin regions) (31 tissues or cell lines; Additional file 1: Table S11). The bottom panel in Fig. 5b shows the peak scores for CTCF-binding sites and DHSs. As expected, we recognized CTCF-binding sites and DHSs in the rs1476679 region. Additionally, we found CTCF-binding sites in the gene body of *PILRA* and *PILRB* and upstream of *PILRA*, *PILRB* and *GATS*. These results suggested that rs1476679 spatially interacts with *PILRA*, *PILRB* and *GATS* via CTCF.

Besides CTCF various other TFs were found to bind to the enhancers in the rs1476679 region and the region 1 kb downstream (Fig. 4a), suggesting that these TFs could act on promoter regions of *PILRA*, *PILRB* or *GATS* located in the rs1476679-interacting regions. To assess this hypothesis, we looked for RNAPII-binding promoter regions upstream of *PILRA*, *PILRB* and *GATS,* in the rs1476679-interacting region, using ChIP-seq data for RNAPII in brain tissues or neuronal cell lines from the ChIP-Atlas database (10 tissues or cell lines, Additional file 1: Table S11). By combining the ChIP-seq data with the DNase-seq data mentioned above, we identified two regions upstream of *PILRB* and *GATS* that included both RNAPII-binding sites and DHSs, indicating that these two regions are active promoter regions in neuronal cell lines (Fig. 5b (bottom panel)). However, we did not find those signals in the region upstream of *PILRA*. The presence of active promoters in two regions upstream of *PILRB* and *GATS* was consistent with estimations based on histone modifications, although a promoter in the region upstream of *PILRA* was ambiguous (Additional file 3: Figure S2). The RNAPII-binding sites of *GATS* and *PILRB* and the interacting regions were 2 kb~ 7 kb apart and did not overlap (Fig. 5b (upper panel) (see Discussion). Taken together, our results suggested that the enhancers near rs1476679 approached the promoter regions of *PILRB* and *GATS* via CTCF-CTCF interactions.

We visualized significant chromatin loops with the region 100 kb downstream of rs1476679 to search for other candidate eQTL genes affected by chromatin loops from rs1476679 (Additional file 3: Figure S3A). We found that rs1476679 interacted with a region within approximately 6 kb of the TSS of the *NYAP1* (neuronal tyrosine-phosphorylated phosphoinositide-3-kinase adaptor 1) gene (Additional file 3: Figure S3B), which showed a strong eQTL effect with rs1476679 (*p*-value = 1.16 × 10^− 11^ in adipose subcutaneous tissues in the GTEx database) and was significantly upregulated in the AD hippocampus (FDR = 1.35 × 10^− 4^) although not in the entorhinal cortex (FDR = 0.294) (Additional file 3: Figure S3C). CTCF binding sites and active promoter peaks were found in the region upstream of *NYAP1* although their peaks did not overlap with the interacting regions (Additional file 3: Figure S3B, bottom panel) (see Discussion). These results suggested that rs1476679 affects *NYAP1* expression via CTCF-CTCF interactions.

Taken together, our results from the chromatin higher-order structure analysis showed that rs1476679 spatially contacted several eQTL genes via chromatin loops and that rs1476679 likely affects *PILRB* and *GATS*, which were reported as the eQTL genes of rs1476679 in previous studies, through enhancer-promoter interactions. These enhancer-promoter interactions were supported by bindings of various TFs near the rs1476679 region and bindings of RNAPII in upstream of *PILRB* and *GATS*.

### The impact of rs7364180 on many of its eQTL genes may be indirect

Finally, we used a similar analysis to identify chromatin loops formed by rs7364180. We found that rs7364180 significantly interacted with *CCDC134* and its adjacent genes *MEI1* and *SREBF2* (Additional file 3: Figure S4A); however, no long-range chromatin interactions with the other eQTL genes were identified. These results suggested that rs7364180 does not directly influence the expression levels of most of its eQTL genes. However, *SREBF2* showed strong eQTL effects with rs7364180 in several brain tissues (Additional file 3: Figure S4B). To examine the genes that are regulated by *SREBF2*, whose product is a TF, we used TRRUST, which is a TF-target interaction database based on text mining and manual curation [53]. This analysis showed that *SREBF2* regulates 20 genes that are significantly associated with AD (FDR = 1.60 × 10^− 6^) (Additional file 1: Table S12 and S13). Therefore, many of the eQTL genes identified for rs7364180 may be indirectly affected by the change in *SREBF2* expression.

## Discussion

Previous GWASs have found AD-candidate SNPs, however, how these AD SNPs act to the pathogenesis is little known. In this study, we attempted to uncover those functions, considering epigenetic effects from chromatin higher-order structure. We confirmed our hypothesis that many non-coding AD SNPs are located in enhancers and affected the expression levels of surrounding genes. We also investigated chromatin higher-order structure with the aim of revealing direct interactions between the AD SNPs and eQTL genes through TCC experiments. We report the following findings: (1) nearly 30% of non-coding AD SNPs are located in enhancers; (2) the eQTL genes of the non-coding AD SNPs within enhancers are associated with Aβ formation, synaptic transmission, immune responses, and AD status; (3) rs1476679 and rs7364180 are associated with a particularly large number of eQTL genes; and (4) rs1476679 spatially interacts with many eQTL genes via chromatin loops.

We showed that the DEGs in the cerebellum were not significantly overlapped with the eQTL genes in the independent two datasets (Table 2). The cerebellum in AD does not present neurofibrillary tangles, which are intracellular aggregations of hyperphosphorylated tau protein [54] and has ever been often used as a reference brain region in AD studies [55, 56]. However, recent studies have shown that the cerebellum is influenced by AD pathologies. Studies of functional MRI have reported network-based degeneration in the cerebellum of AD patients [57, 58]. Additionally, a comprehensive proteome study suggested that the cerebellum is affected by different pathways compared to the other brain regions [59]. Our above result may reflect the specificity of the cerebellum.

Our findings revealed that rs1476679 is not only found in the enhancer but also directly interacts with eQTL genes through chromatin loops. In addition to *PILRB* and *GATS*, which were reported in previous studies [15, 52], we found *NYAP1* to be a candidate eQTL gene affected by rs1476679 via a chromatin loop. *NYAP1* regulates neuronal morphogenesis [60]. A recent large-scale GWAS of AD identified an SNP around *NYAP1* [10] and we found *NYAP1* to be upregulated in the AD hippocampus. Thus, *NYAP1* may be related to AD pathology.

Our TCC experiments showed that rs7364180 interacts with *CCDC134* and its adjacent genes, *MEI1* and *SREBF2*. Although chromatin interactions with other eQTL genes were not identified, we found that *SREFB2*, which is a TF, regulates the expression of 20 genes significantly associated with AD. In addition, previous studies have shown that *SREBF2* controls brain cholesterol synthesis and is involved in diabetes, which is associated with an increased risk of AD [61, 62], and that AD model mice overexpressing *SREBF2* show Aβ accumulation and neurofibrillary tangle formation [63]. Overall, these findings suggest that rs7364180 might exert its effect on AD-associated genes, at least in part, indirectly via *SREBF2*.

rs1476679 and rs7364180 are located in CTCF-binding sites. CTCF is a regulator of chromatin topology that regulates the boundaries of TADs [43, 44, 64, 65]. Mutations in CTCF-binding sites are associated with diseases [19, 66]. For instance, in frontotemporal lobar degeneration, which belongs to the group of neurodegenerative diseases that includes AD, a SNP in a CTCF-binding site modifies the surrounding chromatin conformation and spatially regulates the expression level of a causative gene, *TMEM106B*, leading to neuronal death [66]. These reports support the hypothesis that the disease risk associated to rs1476679 and rs7364180 are due to epigenetic effects occurring via chromatin loops.

We found 19 SNPs in enhancer regions for which TF binding was confirmed by ChIP-seq data and that were associated with at least one eQTL gene (Table 3). Of them, rs4147929 in the *ABCA7* intron was identified through IGAP GWAS [8]. The enhancer including the *ABCA7* locus was activated in immune cells, such as monocytes, B cells, and T cells. *ABCA7* is highly expressed in human monocytes that induced into macrophages [67]. Additionally, the expression level of *ABCA7* is also high in human microglia [68]. The monocytes-derived macrophages and microglia response to immune responses and have phagocytic activities. The epigenetic data that we used in this study did not include them from microglia, however, epigenetic status between the monocytes and microglia may similar. This suggests that the *ABCA7* locus rs4147929 could have the eQTL effects in microglia and could affect pathology in brain regions.

Our study has several limitations. First, the interactions between the SNP and the eQTL genes were shown using only the TCC method. Second, the RNAPII-binding sites of *GATS*, *PILRB*, and *NYAP1* did not overlap with the interacting regions. In TCC, an interacting DNA pair is fragmented with a restriction enzyme and then becomes a chimeric DNA fragment after ligation. Both ends of this fragment are sequenced by pair-end sequencing. The results from pair-end sequencing show the proximal region that spatially contacted each other. Therefore, interacting regions are concentrated on the cut site of a restriction enzyme and do not necessarily overlap with regulatory regions such as promoters. We must analyze more precise chromatin interactions to prove our results.

## Conclusions

In conclusion, multi-omics data analyses, including analyses of histone modifications, eQTL associations, protein binding, and chromatin higher-order structure data, suggested mechanisms by which non-coding AD SNPs identified in AD GWASs may confer disease risk. The main novel finding of this investigation is the eQTL mechanisms identified between rs1476679 at the *ZCWPW1* locus and its eQTL genes through chromatin interaction analysis. In future studies, we need to compare postmortem human brains from AD patients with those of normal healthy individuals to clarify the details of chromatin higher-order structure in AD.

## Additional files

Additional file 1: **Tables S1** List of 392 non-coding AD SNPs. **Table S2** List of 127 tissues or cell types. **Table S3** List of 25 chromatin states from ChromHMM. **Table S4** Gene functional enrichment analysis using DAVID database. **Table S5** Gene functional enrichment analysis using the MSigDB database. **Table S6** Differentially expressed genes (DEGs) between AD and non-AD in each brain tissue in each dataset. **Table S7** List of neuronal tissues or cell types in which 19 SNPs are located in TF-binding sites from ENOCDE ChIP-seq data. **Table S8** List of tissues or cell types in which AD candidate gene loci showed enhancer activity. **Table S9** List of tissues or cell types in which rs1476679 or rs7364180 loci showed enhancer activity. **Table S10** List of tissues or cell types in which rs1476679 or rs7364180 loci are located in CTCF-binding sites from ChIP-seq data in UCSC genome browser. **Table S11** ChIP-seq experiments recorded in ChIP-Atlas. **Table S12** Diseases associated with SREBF2. **Table S13** SREBF2 targets from TRRUST. (XLSX 71 kb)

Additional file 2: Supplementary information. (DOCX 32 kb)

Additional file 3: **Figure S1** Higher-order chromatin structure of the rs1476679-containing region as assessed by chromatin interaction analysis by paired-end tag sequencing (ChIA-PET) experiments. **Figure S2** Upstream regions of *GATS* and *PILRB* genes show prominent promoter activity as estimated from histone modifications. **Figure S3** Chromatin interactions between the rs1476679 locus and *NYAP1*. **Figure S4** Higher-order chromatin structure of the rs7364180-containing region. (DOCX 1071 kb)

## Data Availability

The datasets used and/or analyzed during the current study are available from the corresponding author on reasonable request.

## Abbreviations

AD SNP: AD-associated SNP
AD: Alzheimer’s disease
CTCF: CCCTC-binding factor
DFG: Differentially expressed gene
DHS: DNase I-hypersensitive site
eQTL: expression quantitative trait locus
GWAS: Genome-wide association study
LOAD: Late-onset Alzheimer’s disease
RNAPII: RNA polymerase II
SNPs: Single-nucleotide polymorphisms
TAD: Topologically associated domain
TCC: Tethered conformation capture
TF: Transcription factor
