# Supplementary material for: Enhancer variants associated with Alzheimer’s disease affect gene expression via chromatin looping
Source: BMC Med Genomics. 2019 Sep 9;12:128. doi: 10.1186/s12920-019-0574-8 (PMC6734281; doi:10.1186/s12920-019-0574-8)
Supplement: Supplementary file 2 — Supplementary information. (DOCX 32 kb) [file 12920_2019_574_MOESM2_ESM.docx]

**Supplementary Information**

**Cell culture**

　We used two cell lines for this study; a neuroblastoma cell line, SK-N-SH (American Type Culture Collection, Manassas, VA, USA) (HTB-11), and an astrocytoma cell line, U-251MG (Japan Collection of Research Bioresources Cell Bank, Ibaraki, Osaka, Japan) (IFO50288). SK-N-SH was grown in Eagle's MEM medium supplemented with 10% FBS, 1 mM sodium pyruvate, 0.1 mM each non-essential amino acids and antibiotics (Gibco(R) Penicillin-Streptomycin, 10,000 U/mL (Life Technologies)). U-251MG was grown in Eagle's MEM medium supplemented with 10% FBS and antibiotics. Both cell lines were cultured at 37°C with 5% CO_2_.

**TCC library preparation**

A TCC library was prepared according to a method reported by Kalhor *et al.* with minor modifications [1]. The cells were cross-linked with 2 % formaldehyde on 10 minutes incubation, and then 2 M glycine was added to a final concentration of 125 mM to stop the reaction. Since cells stuck strongly to the culture dish during the crosslinking reaction, which made it hard to harvest them, they were washed twice using PBS(-) and then treated with trypsin-EDTA (0.25% trypsin and 1 mM EDTA, Invitrogen) for 15 minutes at 37 °C. The dishes were chilled on ice and the cells were harvested by pipetting on ice to avoid proteolysis. To isolate nuclei, cells were suspended in 50 mL of ice-cold lysis buffer (10 mM Tris-HCl, pH 8.0, 10 mM NaCl, 1 % IGEPAL CA-630 (Sigma), and 1/500 (vol/vol) protease inhibitor cocktail (Nacalai Tesque)), stirred for 90 min at 4 °C and then centrifuged at 1,600× g [2,3]. Nuclei were suspended in wash buffer 1 (50 mM Tris-HCl, pH 8.0, 50 mM NaCl and 1 mM EDTA). The suspension was incubated for 10 minutes at 65 °C after the addition of 95 µL 2 % SDS. After that, 105 µL 25 mM iodoacetyl-PEG2-biotin (IPB) (Thermo Fisher Science) was added, followed by incubation for 40 minutes at room temperature to biotinylate cysteine residues. Chromatin was digested with *Hin*dIII (New England Biolabs) overnight at 37 °C with rotation. After digestion, the mixture was placed in a Slide-A-Lyzer Dialysis Cassette (20K MWCO) (Pierce Protein Research Products, Rockford, Illinois) and dialyzed for four hours at room temperature against 1 L of the TE buffer (10 mM Tris-HCl, pH 8.0, 1 mM EDTA) to remove IPB remaining from the biotinylation step. The TE buffer was renewed after three hours.

　Digested chromatin was immobilized on 0.4 mL Dynabeads(R) MyOne Streptavidin T1 beads (Invitrogen) to remove non-crosslinked DNA fragments. The magnetic beads were treated with neutralized IPB and then washed twice with wash buffer 2 (10 mM Tris-HCl, pH 8.0, 50 mM NaCl, 0.4 % Triton X-100). The DNA ends were labeled by adding 0.7 µL 10 mM dATP, 0.7 µL 10 mM dTTP, 0.7 µL 10 mM dGTPalphaS (AXXORA), 15 µL 0.4 mM biotin-14-dCTP (Invitrogen), and 5 µL 5 units/µL Klenow (New England Biolabs), and then incubated at room temperature for 40 min. The labeling reaction was stopped by adding 5 µL 0.5 M EDTA, and then the beads were washed twice and resuspended in 500 µL of wash buffer 3 (50 mM Tris-HCl, pH 7.4, 0.4 % Triton X-100, 0.1 mM EDTA). For ligation, the beads suspension was added to 4.4 mL of ligation mix (containing 180 µL 10 % Triton X-100, 250 µL 10× ligase buffer (New England Biolabs), 100 µL 1 M Tris-HCl, pH 7.4, and 50 µL 100× BSA (New England Biolabs)). After the addition of 2 µL DNA ligase (New England Biolabs), the suspension was gently rocked on a reciprocal shaker at 16 °C. After 4 hours incubation, 0.2 mL 0.5 M EDTA was added to stop the ligation step. To purify the ligated DNA, reverse-crosslinking and protein digestion with proteinase K (New England Biolabs) were performed overnight at 65 °C. DNA free from streptavidin-coated beads was purified by phenol-chloroform-isoamylalcohol extraction. To determine whether or not the ligatin step had been successful, PCR amplification was performed using the primers described by Lieberman-Aiden *et al.* [4], and then the PCR products were sequenced using a Sanger sequencer ABI 3130 (Applied Bioscience).

After quality checking, biotinylated residues from unligated DNA ends were removed using *Escherichia coli* exonuclease III (New England Biolabs). The DNA was sheared with an acoustic solubilizer Covaris S2 (Covaris Inc.), and the fragment size was checked with a 2100 Bioanalyzer (Agilent Technologies). The biotin-labeled DNA fragments were pulled-down using 10 µL streptavidin-coated magnetic beads (Dynabeads(R) MyOne Streptavidin C1 beads (Invitrogen)). The DNA fragments were ligated with paired-end sequencing adaptors (Illumina, San Diego, CA) and then amplified (12-15 cycles). After amplification, the library was size-selected by agarose gel electrophoresis to remove DNA fragments whose length was less than 350 bp or more than 500 bp. The DNA fragments of 350-500 bp in the agarose gel were purified with a QIAquick gel extraction kit (QIAGEN). The size and concentration of the purified library were determined with a 2100 Bioanalyzer.

**Processing of the sequencing output**

Image analysis and base calling were performed using Illumina RTA and CASAVA with a default parameter. Mapping and filtering were carried out according to a procedure reported by Imakaev *et al.* [5]. In brief, sequenced reads were mapped to the human reference genome (hg19) using Bowtie2 [6]. The reads of 25 bp were mapped at first. If the reads were not mapped uniquely, they were extended to 30 bp and re-mapped. This extension and re-mapping process was repeated until the read length of 75bp was reached. To remove non-informative pairs, the following read pairs were removed: (1) self circles (both reads mapped in the same restriction fragment), (2) dangling ends (sum of the distances between read and corresponding *Hin*dIII recognition site more than 500 bp), and (3) redundant (which may result from PCR amplification). We used a library “hiclib” (provided by the Leonid Mirny laboratory (https://bitbucket.org/mirnylab/)) for these filtering steps. The processing steps described above were carried out with our in-house computational pipeline.

**Expression quantitative trait loci (eQTL)**

　The GTEx Portal database provides SNPs associated with gene expression from 449 individuals not restricted to specific diseases or conditions in 44 diverse postmortem tissues. RNA expression was measured by RNA sequencing. Genotyping was performed on the Illumina Human Omni 2.5M and 5M Beadchips.

　The BRAINAC database provides SNPs associated with gene expression from 134 neuropathologically normal individuals in 10 postmortem brain regions: cerebellar cortex, frontal cortex, hippocampus, inferior olivary nucleus (sub-dissected from the medulla), occipital cortex, putamen (at the level of the anterior commissure), substantia nigra, temporal cortex, thalamus (at the level of the lateral geniculate nucleus), and intralobular white matter. The BRAINAC database was calculated eQTL effects in the 10 brain regions and the average across all available regions. RNA expression was measured using an Affymetrix Exon 1.0 ST array. Genotyping was performed on the Illumina Infinium Omni1-Quad BeadChip.

**Publicly available gene expression datasets**

　syn5550404 includes gene expression data for 159 cerebellum (CBE) and 160 temporal cortex (TC) samples from North American Caucasian subjects with neuropathological diagnosis of AD (n=82) or elderly controls without neurodegenerative diseases (n=77 in CBE; n=78 in TC). All subjects were from the Mayo Clinic Brain Bank (MCBB) or the Banner Sun Health Research Institute. All ADs had definite diagnosis according to the the National Institute of Neurological and Communicative Disorders and Stroke and the Alzheimer's Disease and Related Disorders Association (NINCDS-ADRDA) criteria and had Braak neurofibrillary tangle (NFT) stage of IV or greater. Control subjects had Braak NFT stage of III or less, the Consortium to Establish a Registry for Alzheimer's Disease (CERAD) neuritic and cortical plaque densities of 0 (none) or 1 (sparse) and lacked any of the following pathologic diagnoses: AD, Parkinson’s disease, dementia with Lewy bodies, vascular dementia, progressive supranuclea palsy, motor neuron disease, corticobasal degeneration, Pick’s disease, Huntington’s disease, frontotemporal lobar degeneration, hippocampal sclerosis or dementia lacking distinctive histology. Gene expression measures were generated, using next-generation RNA sequencing with Illumina HiSeq 2000 sequencers.

　In GSE5281, brain samples were collected at three Alzheimer’s Disease Centers (Washington University, Duke University, and Sun Health Research Institute). Individuals clinically classified as neurologically normal (10 males and 4 females) with a mean age of 79.8 ± 9.1 yr. Clinically classified late-onset AD-afflicted individuals (15 men and 18 women) with a mean age at death of 79.9 ± 6.9 yr. Samples were collected (mean postmortem interval (PMI) of 2.5 h) from six brain regions that are either histopathologically or metabolically relevant to AD and aging; these include the entorhinal cortex (BA 28 and 34), superior frontal gyrus (BA 10 and 11 and approximate BA 8), hippocampus, primary visual cortex (BA 17), middle temporal gyrus (BA 21 and 37 and approximate BA 22), and the posterior cingulate cortex (BA 23 and 31). Each brain tissues were analyzed on an Affymetrix Human Genome U133 Plus 2.0 Array.

　In GSE44770, 230 autopsied tissues from dorsolateral prefrontal cortex (PFC), visual cortex (VC) and cerebellum (CR) in brains of LOAD patients, and non-demented healthy controls, collected through the Harvard Brain Tissue Resource Center (HBTRC), were profiled on a custom-made Agilent 44K array. All subjects were diagnosed at intake and each brain underwent extensive LOAD-related pathology examination. Gene expression analyses were adjusted for age and sex, PMI in hours, sample pH and RNA integrity number (RIN). In the overall cohort of LOAD and non-demented brains the mean ± SD for sample PMI, pH and RIN were 17.8±8.3, 6.4±0.3 and 6.8±0.8, respectively.

**Supplementary References**

1. Kalhor, R., Tjong, H., Jayathilaka, N., Alber, F. and Chen, L. (2012) Genome architectures revealed by tethered chromosome conformation capture and population-based modeling. *Nature Biotechnology*, **30**, 90-98.
2. Liu, J. and Francke, U. (2006) Identification of cis-regulatory elements for MECP2 expression. *Human Molecular Genetics*, **15**, 1769-1782.
3. Murrell, A., Heeson, S. and Reik, W. (2004) Interaction between differentially methylated regions partitions the imprinted genes Igf2 and H19 into parent-specific chromatin loops. *Nature Genetics*, **36**, 889-893.
4. Lieberman-Aiden, E., van Berkum, N.L., Williams, L., Imakaev, M., Ragoczy, T., Telling, A., Amit, I., Lajoie, B.R., Sabo, P.J., Dorschner, M.O. *et al.* (2009) Comprehensive mapping of long-range interactions reveals folding principles of the human genome. *Science*, **326**, 289-293.
5. Imakaev, M., Fudenberg, G., McCord, R.P., Naumova, N., Goloborodko, A., Lajoie, B.R., Dekker, J. and Mirny, L.A. (2012) Iterative correction of Hi-C data reveals hallmarks of chromosome organization. *Nature Methods*, **9**, 999-1003.
6. Langmead, B. and Salzberg, S.L. (2012) Fast gapped-read alignment with Bowtie 2. *Nature Methods*, **9**, 357-359.
