# Supplementary material for: Enhancer variants associated with Alzheimer’s disease affect gene expression via chromatin looping
Source: BMC Med Genomics. 2019 Sep 9;12:128. doi: 10.1186/s12920-019-0574-8 (PMC6734281; doi:10.1186/s12920-019-0574-8)
Supplement: Supplementary file 3 — Figure S1 Higher-order chromatin structure of the rs1476679-containing region as assessed by chromatin interaction analysis by paired-end tag sequencing (ChIA-PET) experiments. Figure S2 Upstream regions of GATS and PILRB genes show prominent promoter activity as estimated from histone modifications. Figure S3 Chromatin interactions between the rs1476679 locus and NYAP1. Figure S4 Higher-order chromatin structure of the rs7364180-containing region. (DOCX 1071 kb) [file 12920_2019_574_MOESM3_ESM.docx]

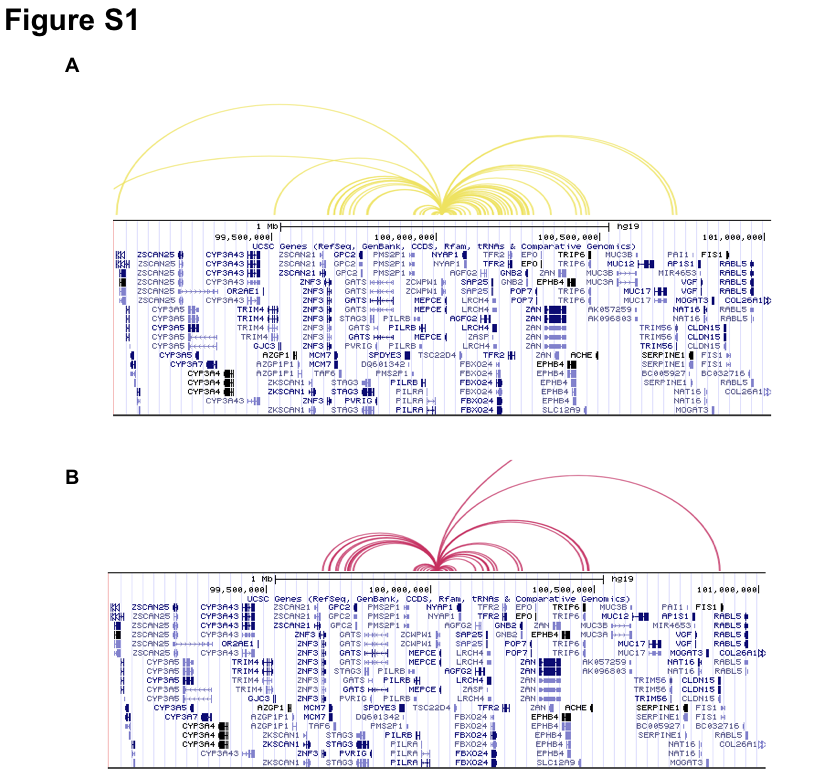


**Figure S1. Higher-order chromatin structure of the rs1476679-containing region as assessed by chromatin interaction analysis by paired-end tag sequencing (ChIA-PET) experiments**

Each curved line represents chromatin loops associated with RNA polymerase II in K562 (A) and MCF-7 (B) cell lines. These figures were modified from the 3D Genome Browser (http://promoter.bx.psu.edu/hi-c/index.html).


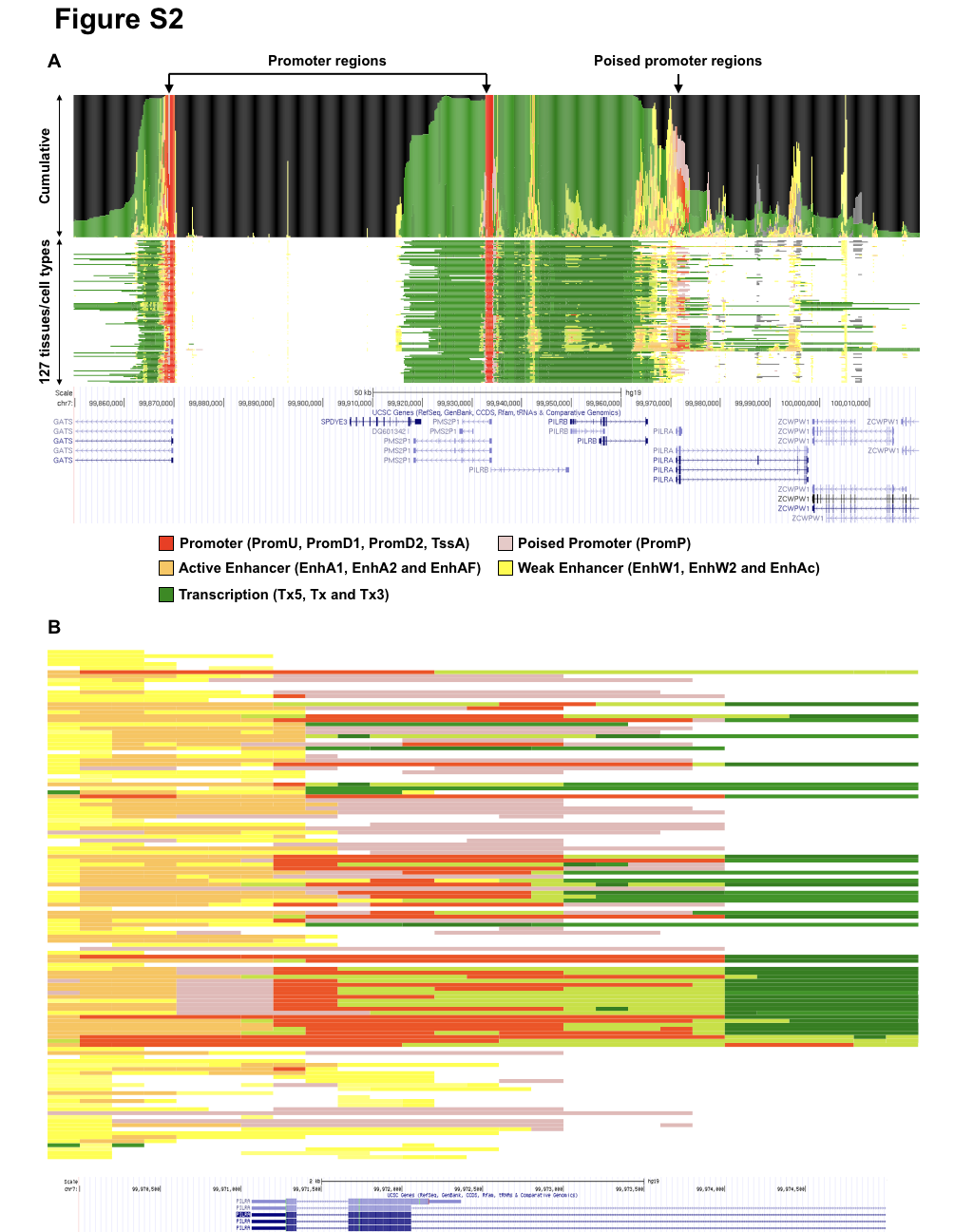


**Figure S2. Upstream regions of *GATS* and *PILRB* genes show prominent promoter activity as estimated from histone modifications.**

(A) Five representative chromatin state groups are shown (see color key). The chromatin state names are shown in parentheses (see detail in Materials and Methods). Details of all 25 chromatin state names are given in Additional file 1: **Supplement Table S3**. (B) The chromatin groups in a promoter region of *PILRA* gene.


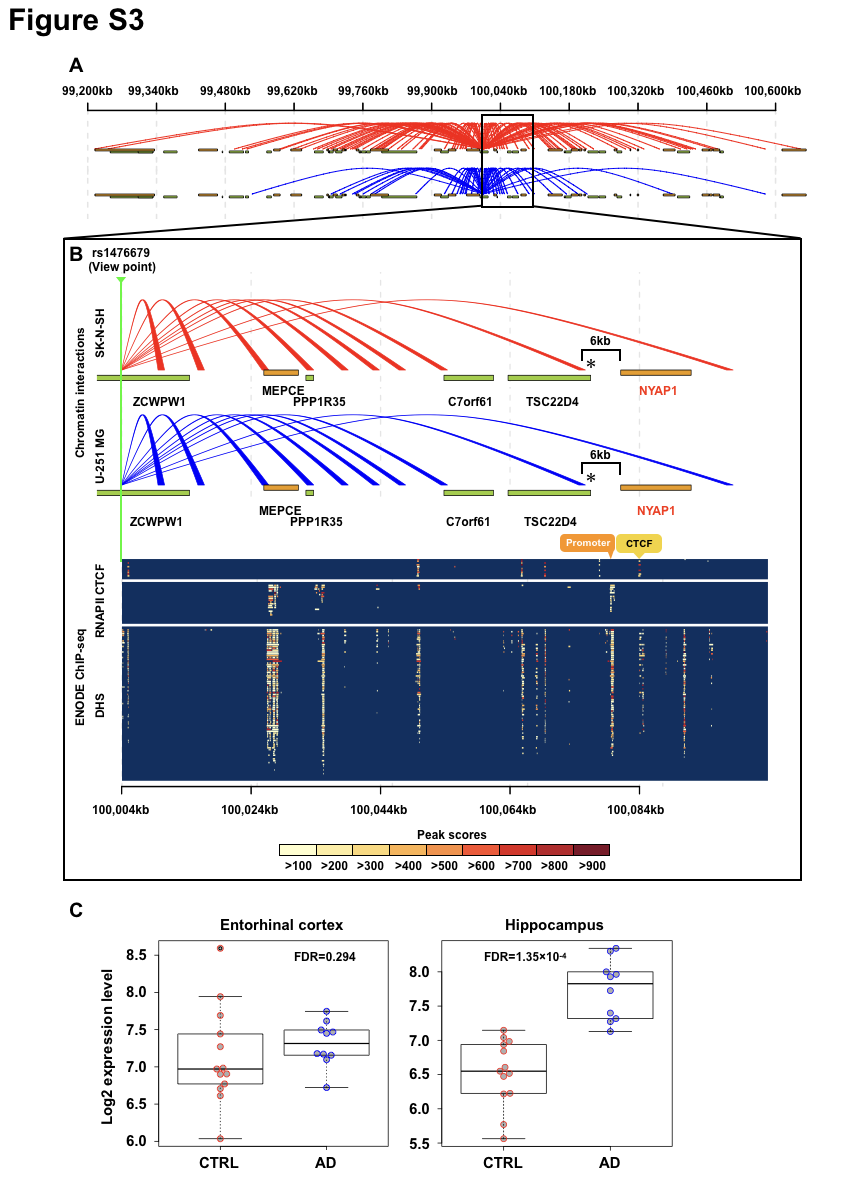


**Figure S3. Chromatin interactions between the rs1476679 locus and *NYAP1***

(A) Chromatin interactions from rs1476679 locus as determined by TCC experiments. Red and blue lines represent statistically significant chromatin interactions from rs1476679 in SK-N-SH and U-251 MG, respectively. (B) Zoom-in region of rs1476679 locus. Upper panel indicates chromatin interactions from s1476679 locus. Orange and green bands indicate gene bodies on the positive and negative strands, respectively. Gene symbols in red indicate eQTL genes of rs1476679. Asterisks indicate chromatin interactions from the rs1476679 locus to around the TSS of *NYAP1*. In the bottom panel, a color plot indicates peak scores from ChIP-seq data for CTCF or RNA polymerase II (RNAPII) and DNase-seq data to show DNase I hypersensitive sites (DHSs). Each row in the color plot represents different neuronal cell lines (18 experiments (rows) including nine tissues or cell lines in CTCF ChIP-seq; 21 experiments including ten cell lines in RNAPII ChIP-seq; 82 experiments including 31 cell lines in DNase-seq; Additional file 1: **Supplement Table S11**). (C) Expression level of *NYAP1* in hippocampus and entorhinal cortex from GSE5281. Boxes represent the interquartile range between the first and third quartiles and median (internal line). Whiskers denote the lowest and highest values within 1.5 times the range of the first and third quartiles, respectively; dots represent *GATS* and *PILRB* expression levels in each sample.


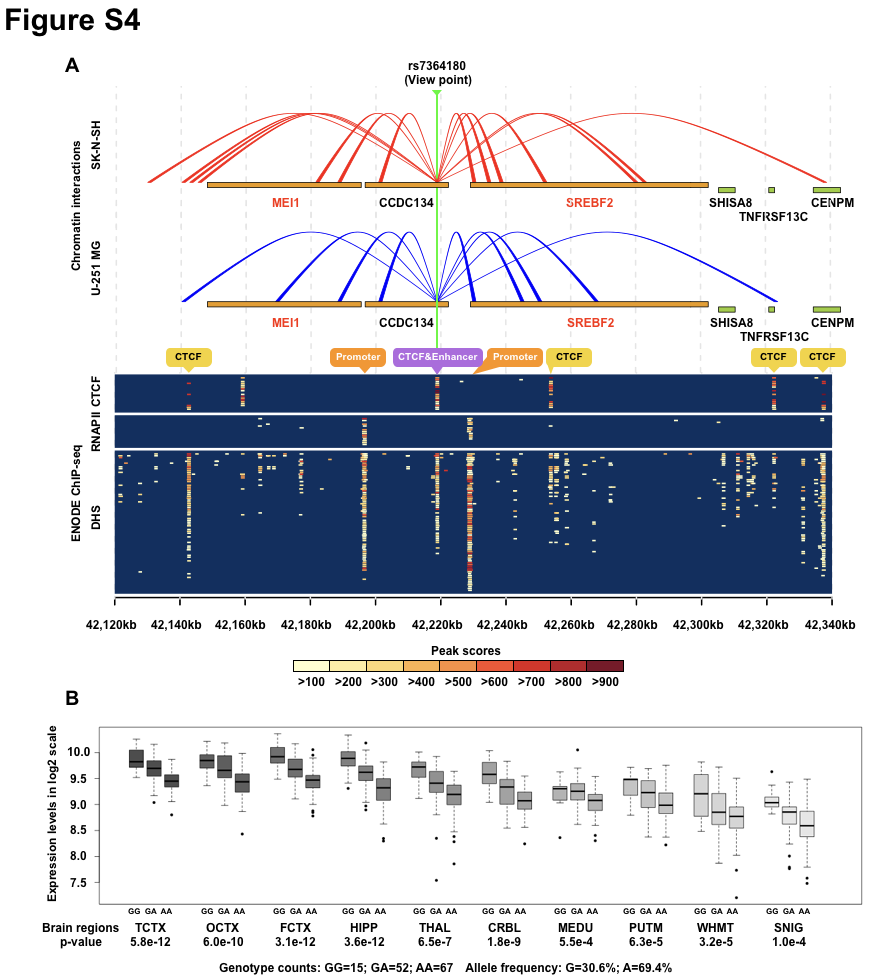


**Figure S4. Higher-order chromatin structure of the rs7364180-containing region**

(A) Chromatin interactions from the rs7364180 locus as determined by TCC experiments and protein binding in the corresponding region. In the upper panel, red and blue lines represent statistically significant chromatin interactions from rs7364180 in SK-N-SH and U-251 MG, respectively. Orange and green bands indicate gene bodies on the positive and negative strands, respectively. Gene symbols in red indicate eQTL genes of rs7364180. In the bottom panel, a color plot indicates peak scores from ChIP-seq data for CTCF or RNA polymerase II (RNAPII) and DNase-seq data to show DNase I hypersensitive site (DHS). Each row in the color plot represents different neuronal cell lines (18 experiments (rows) including nine tissues or cell lines in CTCF ChIP-seq; 21 experiments including ten cell lines in RNAPII ChIP-seq; 82 experiments including 31 cell lines in DNase-seq; Additional file 1: **Supplement Table S11**). (B) eQTL associations between rs7364180 genotypes (GG, GA, and AA) and *SREBF2* expression levels in the following ten brain tissues: TCTX, temporal cortex; OCTX, occipital cortex (specifically the primary visual cortex); FCTX, frontal cortex; HIPP, hippocampus; THAL, thalamus; CRBL, cerebellar cortex; MEDU, medulla (specifically the inferior olivary nucleus); PUTM, putamen; WHMT, intralobular white matter; and SNIG, substantia nigra. These box plots were modified from the BRAINEAC database.
